# What Can We Change in Diet and Behaviour in Order to Decrease Carotid Intima-Media Thickness in Patients with Obesity?

**DOI:** 10.3390/jpm11060505

**Published:** 2021-06-03

**Authors:** Anna Maria Rychter, Dariusz Naskręt, Agnieszka Zawada, Alicja Ewa Ratajczak, Agnieszka Dobrowolska, Iwona Krela-Kaźmierczak

**Affiliations:** 1 Department of Gastroenterology, Dietetics and Internal Diseases, Poznan University of Medical Sciences, 60-355 Poznań, Poland; aga.zawada@gmail.com (A.Z.); alicjaewaratajczak@gmail.com (A.E.R.); agdob@ump.edu.pl (A.D.); krela@op.pl (I.K.-K.); 2 Department of Internal Medicine and Diabetology, Poznan University of Medical Sciences, 60-834 Poznań, Poland; dnaskret@poczta.onet.pl

**Keywords:** intima-media thickness, obesity, cardiovascular risk, atherosclerosis

## Abstract

Atherosclerosis—considered the major cause of cardiovascular diseases (CVDs)—is strongly associated with obesity, to which it strongly contributes. Moreover, atherosclerosis is characterised by a long asymptomatic phase, and its progression can lead to serious cardiovascular (CV) events. The carotid intima-media thickness (cIMT) has been determined as a predictor of CV events, as well as a marker of subclinical atherosclerosis, and has been used in clinical trials as an alternative assessment method or a surrogate endpoint. It should be noted that several behavioural approaches can directly influence the cIMT values, and decrease or increase the CV risk. In our paper, we aimed to summarize the current knowledge regarding IMT measurement among patients with obesity as a risk group—also in terms of the obesity paradox where the diagnosis of subclinical atherosclerosis is especially essential and implements the early therapeutic approach. We also summarized behavioural, modifiable factors, such as the Mediterranean diet, the Dietary Approach to Stop Hypertension Diets, body weight reduction or the intake of micro- and macronutrients, with a particular focus on the studies where the cIMT values were one of the outcomes. In order to collect the literature data related to the presented topic, the PubMed database was explored.

## 1. Introduction

**Figure 1:**
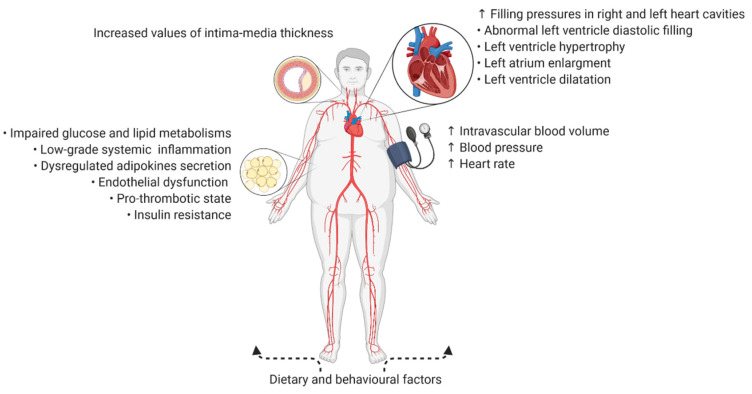
Indirect and direct ways in which obesity increases the cardiovascular risk. ↑—increased.

Atherosclerosis is a chronic inflammatory disease of the arteries, with a long asymptomatic phase, and has been considered the major cause of cardiovascular diseases (CVDs). It is estimated that atherosclerosis constitutes an underlying cause of around 50% of all deaths in the Western countries [1]. Moreover, CVD accounts for 37% and 35% of potential years of life lost (PYLL) among females and males, respectively. In 2017, almost 35 million people suffered from ischaemic heart disease (IHD), which was a major contributor to disability-adjusted life years (DALYs; one DALY corresponds to one lost year of healthy life) due to CVD [2]. Obesity—defined by the World Health Organization as an excessive fat accumulation, with a Body Mass Index (BMI) greater than or equal 30 m/kg^2^—is a major modifiable risk factor of CVD significantly contributing to the development of atherosclerosis [3]. A recently published meta-analysis demonstrated that each 10 cm increase of waist circumference increased the risk of CVD by 4.0% and 3.4% among men and women, respectively [4]. Furthermore, obesity prevalence has been on the increase for a number of years, and it can affect the cardiovascular (CV) risk both directly and indirectly (Figure 1).

In the clinical practice, atherosclerosis can be diagnosed by means of several methods. In the late 20th century, the association between an ultrasound measurement of the aortic wall thickness and atherosclerosis was observed, which further led to a conclusion that hypercholesterolemic patients presented a larger common carotid intima-media thickness (cIMT)—the thickness of the intimal and medial layer of the carotid artery wall [5]. Subsequently, cIMT was presented as a predictor of CV events and a marker of subclinical atherosclerosis in various populations. However, several aspects—for example, various guidelines for measuring cIMT in a given country, or the choice of measurement sites—should be considered when evaluating whether cIMT should be a routine method in the clinical practice. Nevertheless, cIMT is frequently used in clinical trials as an alternative method, or a surrogate endpoint, also among the population suffering from obesity.

Moreover, the relationship between atherosclerosis and obesity is complex, with inflammatory state as the major link [6,7]. Excessive body weight is often associated with a localized inflammation in the adipose tissue, which leads to the low-grade, chronic, systemic inflammation; however, the initial trigger for obesity-associated inflammation has not been fully discovered [8]. Nevertheless, among patients with obesity frequently present with an increased number of macrophages, elevated concentrations of inflammatory mediators—e.g., interleukin-6, interleukin-1β and tumour necrosis factor-α—or an altered secretion of adipokines (characterized by pro- and anti-inflammatory properties) [9]. Furthermore, systemic inflammation induced by obesity is also one of the triggering factors of obesity-associated metabolic comorbidities, e.g., type 2 diabetes mellitus (T2DM) or insulin resistance. It should be highlighted that elements such as insulin resistance and several adipokines (e.g., retinol-binding protein 4 (RBP4) or lipocalin) can also negatively affect the vascular endothelium. Moreover, currently available studies have found their association with the increased cIMT values [10,11,12,13,14].

Although behavioural factors, such as diet or overall lifestyle, constitute the essential and modifiable factors in CVD, a number of patients with obesity fail to follow the guidelines regarding proper nutrition [15]. Moreover, several nutrients or dietary patterns have been found to be associated—positively or negatively—with the cIMT values, which could provide a potential approach to decrease the atherosclerosis risk, if analysed more thoroughly.

In our non-systematic review, we aimed to summarize the current knowledge regarding IMT measurement among patients with obesity as a risk group, in which the diagnosis of subclinical atherosclerosis is especially essential and implements the early therapeutic approach. We also summarized behavioural, modifiable factors essential in decreasing cardiovascular risk, with a particular focus on the studies where the cIMT values were one of the outcomes. In order to collect the literature data related to the presented topic, the PubMed database was explored using the terms: “intima-media thickness”, “obesity”, “bariatric surgery”, “diet”, “physical activity”, “cardiovascular risk”.

## 2. Intima-Media Thickness in General Practice

Usually, symptomatic CVD occurs when atherosclerosis progresses to a flow-limiting disease leading to ischemia, or when an atherosclerotic plaque ruptures or erodes [16]. Although atherosclerosis does not inevitability lead to a CV event, it increases its risk. Therefore, the identification of high-risk, asymptomatic patients will reduce the CVD risk [17]. In order to define the presence of subclinical vascular disease, imaging of the arteries was proposed as one of the risk assessment methods. In fact, effectiveness of this technique was confirmed in the prospective trials with long-term follow up studies or observational studies (e.g., the Multi-Ethnic Study of Atherosclerosis, the Carotid Atherosclerosis Progression Study or the Rotterdam study). Additionally, cIMT has become an established, independent predictor for future cardiovascular events, e.g., stroke or myocardial infarction [18,19,20,21]. However, cIMT measurement should be combined with other methods, for instance plaque measurements, in order to provide the highest prediction for CV events. According to the guidelines of the European Society of Cardiology/European Atherosclerosis Society (ESC/EAS), IMT is a poorer indicator than the coronary artery calcium (CAC) score and carotid plaque detection, although the MESA study found that IMT is a better predictor in terms of stroke. Moreover, IMT should be considered among individuals who present a low or moderate risk—mostly asymptomatic 45–75-year-old men and 55–75-year-old women, as well as in individuals with the metabolic syndrome, family history of premature coronary heart disease and two or more National Cholesterol Education Program (NCEP) risk factors [19,22]. The above-mentioned MESA study also helped clarify several technical aspects of the cIMT measurement process improving the reproducibility and increasing the predictive power for CV events, which can also be found in the statement of the American Society Echocardiography [23]. Moreover, according to the Mannheim Consensus, it is essential to distinguish the difference between cIMT and carotid plaque, which encroaches into the arterial lumen by at least 0.5 mm, or 50% of the surrounding IMT value, or presents a thickness > 1.5 mm as measured from the media–adventitia interface to the intima–lumen interface [24]. Furthermore, IMT measurement should be performed in a location free of plaque, with a clearly visible double-line pattern, which will increase the accuracy and reproducibility of the measurement. Moreover, it should preferably be measured at least 5 mm below the end of the far wall of the common carotid artery, as a measurement taken on the near wall may be less reliable and should be recorded independently from the IMT of the far wall. It is also essential to bear in mind that there are several substantial ethnic and gender-specific differences which should be taken into account in the course of the results interpretation [25,26].

Moreover, the Mannheim consensus concludes that IMT has been suggested to represent an important risk marker, although it does not fulfil the characteristics of an accepted risk factor and there is no need to “treat IMT values”. Nevertheless, on the basis of the current data, it has been established that cIMT constitutes a useful clinical tool, does not entail radiation exposure and, additionally, it is a sensitive, reproducible and a relatively low cost technique allowing for the detection of early-state atherosclerosis. Therefore, it should be further investigated in order to determine whether cIMT will also improve long-term CVD outcomes [27,28].

## 3. Intima-Media Thickness Assessment among Patients with Obesity—Subclinical Atherosclerosis and the Predisposing Factors

**Figure 2:**
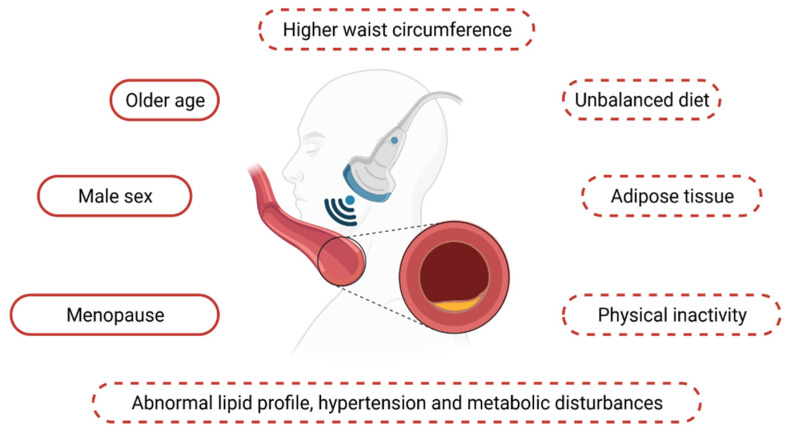
Factors associated with the increased cIMT values. Factors with dashed lines are modifiable and can be changed by behavioural or pharmacological actions.

According to the study by van Mil et al., cIMT was one of two parameters used as an outcome measure for atherosclerosis in patients with morbid obesity, qualified for bariatric surgery [29]. Additionally, cIMT was lower in women when compared to men; however, after the adjustment of co-variables, cIMT was mostly influenced by waist circumference, age, high-density lipoprotein cholesterol (HDL-C) and mean arterial pressure (MAP). Interestingly, low-density lipoprotein cholesterol (LDL-C) levels were higher among women than in men, which could have been influenced by the fact that men were more likely to suffer from T2DM and were prescribed with lipid-lowering drugs. Moreover, postmenopausal women presented higher cIMT values than premenopausal women, 0.591 mm and 0.512 mm, respectively. Men demonstrated higher cIMT values than premenopausal women, which was not statistically significant, whereas the values were higher in postmenopausal women than in men. Although obesity has been one of the main causes of CVD, there is evidence indicating that patients with obesity may actually have a better prognosis for CVD in comparison to patients with a BMI within a normal range. This phenomenon is referred to as the “obesity paradox”, and may be associated with the term “metabolically healthy obesity (MHO)” [30]. According to Romagnolli et al., subjects with obesity, as well as individuals with or without the metabolic syndrome, presented increased subclinical atherosclerosis compared to individuals not suffering from obesity [31]. These differences remained significant following an adjustment for sex, age, race and smoking status in individuals both classified as MHO and as metabolically healthy (MH). Furthermore, an additional adjustment for HDL-C, TG, systolic blood pressure and fasting plasma glucose also did not change the significance between the cIMT values in cases of metabolically unhealthy obesity (MUO) and MHO. These results suggest that obesity is independently associated with an increased cIMT, irrespective of the presence or absence of the metabolic abnormalities. Interestingly, it also suggests that the definition of MHO could be inadequate, as individuals with obesity presented an increased CV risk (defined by the increased cIMT values). Moreover, subclinical atherosclerosis can also correlate with hepatic fat accumulation—in the study by Farcaş et al., fatty liver index (indicating the presence of non-alcoholic fatty liver disease) was strongly associated with cIMT in all obesity phenotypes, including MHO [32]. Jae et al. obtained similar results in their study, where MHO individuals presented a higher prevalence of the subclinical cIMT. However, this association was affected by an increased level of cardiorespiratory fitness levels, which could be considered as a potential modulator of the association between MHO and CVD outcomes [33]. Nevertheless, it should be noted that in this study obesity was defined as BMI ≥ 25 kg/m^2^ (criteria for the Asian population). On the other hand, in the CordioPrev Study, metabolic abnormalities were clear determinants of an increased cIMT, and the presence of an increased body weight increased cIMT, but not when obesity was not associated with a metabolic disease [34]. The values of cIMT in subjects with no CV risk factors were mostly determined by age, male sex, systolic blood pressure (SBP) and LDL-C levels [28]. The association was poorly correlated with SBP and LDL-C level, although it increased significantly for SBP above 120 mmHg and LDL-C levels above 125 mg/dL, which highlights their possible influence on the atherosclerotic process even at non-pathological levels. Moreover, the link between the inflammation and intima-media thickness has been well established. Neutrophil-to-lymphocyte ratio (NRL) in the critically ill patients has been demonstrated to correlate with the systemic inflammation, chronic low-grade inflammation and the atherosclerotic process, e.g., with the accumulation of lipids in the arteries or other atherogenic mechanisms. As Suárez-Cuenca et al. suggested, NRL correlated positively with visceral adiposity and with pro-inflammatory mediators as well as with leptin, and was negatively correlated with adiponectin. As the authors suggested, NRL could constitute a possible biomarker for the subclinical atherosclerosis as it was associated with cIMT higher than 0.9 mm. According to the MESA study, high-sensitivity C-reactive Protein (hsCRP) may be useful in identifying a non-obese risk group of the subclinical atherosclerosis. Interestingly, some gender differences were observed in the association between inflammation and atherosclerosis. Moreover, Lin et al., in their study, claim that hsCRP ≥ 2 mg/L levels were useful in identifying higher values of cIMT among men without obesity, but not in women [35]. The most frequently mentioned factors associated with the increased cIMT values are presented in Figure 2.

## 4. Reducing cIMT Values—Behavioural and Clinical Factors

The cIMT measurement was an independent predictor of a CV event among individuals in the Framingham Offspring Study, conducted by Polak et al. [36]. In the systematic review and a meta-analysis conducted by Lorenz et al., an elevation in cIMT by 0.1 mm, increased the future risk of myocardial infarction by 10–15% and the stroke risk by 13–18% [37]. According to a large, recent meta-analysis, a decrease in the progression of cIMT per 10 µm per year reduced the relative risk for CVD by 0.91 [38]. These results may provide a missing implication of cIMT as a CV risk marker, and a progression of cIMT may be a useful surrogate endpoint in the clinical trials, which could help in the development and assessing the efficacy of new therapeutic approaches.

### 4.1. Diet

Proper diet and nutritional behaviours are essential in both preventive and therapeutic approaches in CVD management. Several dietary patterns and supplementation of the chosen dietary compounds have been effective in decreasing IMT values.

#### 4.1.1. Folate, Folic Acid and Vitamin B12

Increased concentrations of homocysteine have been positively and independently associated with an increased risk of vascular diseases. Folate supplementation (5 mg/day) for 12 weeks had a beneficial effect on the maximum left cIMT values in patients with the metabolic syndrome. However, it did not affect mean values of the left and right cIMT, and the maximum of right cIMT values [39]. Moreover, supplementation of folic acid significantly reduced serum insulin, as well as pro-inflammatory cytokines and improved abnormal lipid profile. Similar results were obtained in other studies—among individuals with at least one CV risk factor, 18 months of folic acid supplementation significantly reduced homocysteine levels and, additionally, a significant regression of cIMT was observed when compared to a significant progression of cIMT in the placebo group [40]. In women suffering from the polycystic ovary syndrome, folate supplementation (5 mg/day) for 8 weeks significantly decreased homocysteine and insulin levels, and improved lipid profile in total-, LDL- and non-HDL-cholesterol concentrations, although it did not affect the other lipid profiles [41]. Moreover, an additional supplementation of folic acid (5 md/day) and vitamin B12 (0.5 mg/day) among individuals with the metabolic syndrome also significantly decreased homocysteine levels, indirectly reducing the CV risk and the atherosclerotic process. In a meta-analysis of randomized clinical trials by Qin et al., the effectiveness of folic acid supplementation in reducing the progression of cIMT was confirmed. This effect was particularly observed in individuals with chronic kidney disease (CKD) or with the high CV risk, where higher reductions of the baseline cIMT and homocysteine concentrations were found [42]. It should be highlighted that patients with CKD are at a significantly higher risk of developing CVD, including ischaemic stroke [43,44]. According to Yu et al., the estimated glomerular filtration rate (eGFR) was negatively correlated with the degree of carotid stenosis [45]. In another study, carotid atherosclerosis was found to be a common pathology of stroke and CKD; moreover, as the authors suggested, IMT could also be a marker for evaluating the pathology of CKD [46]. The relationship between carotid atherosclerosis and CKD could be associated with the chronic inflammation observed in patients with CKD, which could promote the vascular atherosclerosis. Furthermore, among the CKD patients, thyroid dysfunction can also be observed which can increase the CV events risk [47]. Moreover, RBP4—adipokine involved in the atherosclerotic process—was negatively correlated with eGFR in patients with CKD [48]. Strain vessel hypothesis could also constitute the linking mechanism between CKD, CVD and stroke—the strain vessels are branching off directly from the large vessels and can lead to a vascular dysfunction if exposed to a high blood pressure gradient [49].

It is worth bearing in mind that vegetarian diets are mostly cardioprotective—in fact, thinner IMT and better blood pressure, lipid and metabolic profiles were observed among the lacto-vegetarians. Nevertheless, they can be also associated with an increased risk of several nutritional deficiencies, including vitamin B12 deficiencies, which, together with a possible higher salt intake and increased triglyceride levels among individuals following a certain vegetarian diet, can increase the risk of atherosclerosis [50]. Vitamin B12 (500 mcg/day) supplementation for over 24 weeks improved cIMT among the vegetarians; however, this association was subtle, and more research in this aspect is needed [51].

#### 4.1.2. Vitamin D, Vitamin K and Calcium

Vitamins D, K and calcium can beneficially affect atherosclerotic plaque, for instance by improving the insulin status and decreasing pro-inflammatory and oxidative stress markers. Decreased vitamin D levels have been associated with the increased cIMT values among patients with a chronic kidney disease [52]. Moreover, a combined supplementation of vitamin D and calcium improved the metabolic status among women with gestational diabetes mellitus [53]. In the study conducted on T2DM individuals with a coronary heart disease (CHD), a 12-week co-supplementation of the abovementioned compounds (i.e., 5 µg of vitamin D; 90 µg of vitamin K; 500 mg of calcium, twice a day), significantly reduced the maximum levels of the left cIMT, as well as improved the metabolic status. Nonetheless, it did not affect the mean values for the right and left cIMT or the maximum of right cIMT [54]. However, in another study, vitamin D supplementation (combined with the supplementation of omega-3 fatty acids) did not decrease the risk of cardiovascular incidence—such as stroke or myocardial infarction—when compared with the placebo group [55]. As the authors suggested, vitamin D could affect cIMT, although it may not influence the risk of the cardiovascular disease. However, it should be noted that only one dose of vitamin D was tested, and the trial will continue further in order to confirm the results (the results have not been published yet).

**Figure 3:**
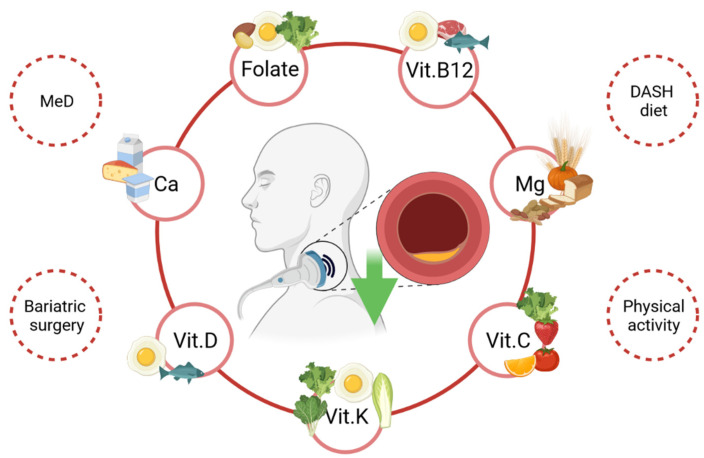
Behavioural and clinical factors that can reduce the cIMT values. MeD—Mediterranean diet; DASH—Dietary Approach to Stop Hypertension; vit.—vitamin; Mg—magnesium. Dietary sources of: calcium (dairy, milk); vit. D (oily fish, eggs); vit. K (green leafy vegetables, eggs), vitamin C (tomatoes, strawberry, green leafy vegetables, citruses); Mg (nuts, wholegrain cereals, pumpkin seeds); vit. B12 (meat, fish, eggs); folate (almonds, green leafy vegetables, eggs).

A single supplementation of vitamin K (90 µg/day) for over 9 months reduced the progression of cIMT and atherosclerosis in individuals with a chronic kidney disease [56]. Moreover, postmenopausal women constitute an interesting group with regard to the cardiovascular risk and calcium supplementation. In terms of an increased risk of osteoporosis following menopause, a proper dietary calcium intake and/or calcium supplementation is essential. However, calcium supplementation among postmenopausal women can have the opposite effects concerning the CV risk and CVD. For instance, after two-year-long supplementation of calcium (800 mg/day), increased cIMT and serum cholesterol concentrations were observed [57]. In several studies, calcium supplementation positively influenced the lipid profile, whereas in other studies, no effect was observed [58,59,60]. In the Reid meta-analysis, calcium supplements increased the risk of myocardial infarction by 27–31% and increased the risk of stroke by 12–20%. In terms of the current data, calcium supplementation should be administered with caution and with the proper assessment of the dietary calcium intake [61]. The dietary sources of calcium, vitamin D and vitamin K are presented in Figure 3.

#### 4.1.3. Magnesium

Magnesium supplementation (250 mg/day) for 24 weeks significantly reduced the mean and maximum levels of the left cIMT, as well as the mean levels of the right cIMT among patients with diabetes and haemodialysis [62]. Moreover, magnesium supplementation improved the insulin and lipid profile statuses, although they did not influence the maximum right cIMT or other metabolic profiles. Patients with diabetes and requiring haemodialysis are a particular group in terms of magnesium status and the atherosclerotic process. In fact, magnesium levels were significantly lower in this group when compared to the controls, and it was associated with an increased risk of atherosclerosis of cIMT [63,64]. Magnesium can improve glucose and lipid profiles, and can positively affect the pro-inflammatory markers [65]. The dietary sources of magnesium are presented in Figure 3.

#### 4.1.4. Mediterranean and the Dietary Approach to Stop Hypertension Diets

Mediterranean diet (MeD) is recommended by the cardiovascular societies in the prevention and treatment of CVD; therefore, adherence to the Mediterranean diet can protect against the development of the atherosclerotic plaque and can decrease overall vascular events [66,67]. According to Gardener et al., MeD was not associated with cIMT; however, the results suggested that the dietary habits consistent with MeD could reduce the carotid atherosclerotic plaque and the stroke risk, since adherence to the MeD was inversely associated with the 75th percentile of plaque thickness, as well as with the 50th percentile of plaque area [68]. In the PREDIMED-Navarra (Prevención con Dieta Mediterránea) study, additional supplementation with olive oil or nuts in MeD did not significantly reduce cIMT after one year of treatment. Nevertheless, these approaches were effective in individuals with the higher baseline IMT values, which could suggest that the subclinical atherosclerotic process can respond to a dietary intervention in a relatively short period of time [69,70]. It should be emphasised that the dietary patterns used in the PREDIMED study demonstrated a reduction of the CVD risk by about 30% [71,72,73,74]. Currently, due to several controversies concerning the PREDIMED study, a new trial known as PREDIMED-PLUS has been conducted, and the final results have been progressively published. Among the individuals with newly diagnosed type 2 diabetes, MeD has been associated with a decrease in the cIMT values, as well as in the circulating levels of the endothelial progenitor cells, and has been more effective in the prevention of the subclinical atherosclerosis progression than the low-fat diet [75,76]. In the MeD, much attention is devoted to the consumption of red, dry wine. Da Luz et al. observed that there were no differences in the IMT values among the red wine drinkers or non-drinkers; however, calcium scores—associated with arterial calcification—were higher among the drinkers [77].

The Dietary Approach to Stop Hypertension (DASH) is a dietary pattern similar to the MeD, with less emphasis on the use of extra-virgin olive oil than in MeD. Additionally, the DASH diet is also useful in reducing the CV risk and is a widely accepted nutritional approach [78,79]. According to Maddock et al., long-term adherence to the DASH diet was associated with decreased cIMT values (even following the adjustment for BMI, smoking status or the physical activity level) [80].

#### 4.1.5. Fats and Carbohydrates

The cIMT values significantly improved following the introduction of vegetable oils (rich unsaturated fatty acids), particularly in the group of flaxseed and olive oils [81]. Moreover, the supplementation of unsaturated fatty acids from vegetable oils improved the inflammatory status and prothrombotic conditions, including reductions in CRP, Apolipoprotein B/Apolipoprotein A1 concentrations—in olive and sunflower oils. Those results are in accordance with numerous guidelines of cardiovascular societies, since it is generally recommended to increase consumption of saturated and trans fatty acids in favour of the unsaturated fatty acids. However, as Angerer et al. pointed out in their study, no reduction of the atherosclerotic plaque in carotid arteries was observed after two-year-long supplementation of unsaturated fatty acids (1.65 g of omega-3 polyunsaturated fatty acids) [82]. Furthermore, it could also be suggested that saturated fat intake is not an independent risk factor for CVD; however, an increased intake of polyunsaturated fatty acids (in replacing saturated or trans fatty acids or carbohydrates) can lower the CV risk [83]. When comparing a low-carbohydrate diet to a traditional diabetic diet in poorly controlled T2DM individuals, no positive changes in the cIMT values were observed (the cIMT values remained relatively the same). Nevertheless, an increase in cIMT was observed among individuals following the second type of diet after 18 months of intervention (the amount of carbohydrates was 82.3 g/day and 161.6 g/day, respectively) [84]. A low-carbohydrate diet had, however, a positive impact on SBP/DBP (diastolic blood pressure), hip and waist circumference and glycaemic control, and did not negatively influence the lipid profile or creatinine levels.

### 4.2. Lifestyle, Physical Activity and Diet Quality Interventions

A greater diet quality (with reference to the consumption of carbohydrates, magnesium, fibre, sugars and the Alternate Health Eating Index) was associated with a greater cIMT regression following a two-year intervention among individuals with type 1 and type 2 diabetes [85]. Similar results were demonstrated when individuals with type 1 and type 2 diabetes increased their intake of fruits and vegetables, and slightly increased the consumption of yoghurt (a planned increase of dairy intake was not achieved). An improvement in the dietary quality was observed, and although it was not fully maintained after 12 months of an intervention, it was associated with a greater reduction of cIMT progression [86]. The cIMT values were inversely associated with the intake of pulses, carbohydrates, cruciferous vegetables, an increased amount of vegetable nitrate and fruits, as well as with a lower intake of total and saturated fats [87,88,89,90].

Lycopene and lutein supplementation significantly decreased the cIMT values following 12 months, whereas the combination of lutein and lycopene (lutein 20 mg/day and lycopene 20 mg/day) was more effective than the supplementation of only lutein (20 mg/day) in reducing cIMT in individuals with subclinical atherosclerosis—by 0.073 mm and 0.035 mm, respectively [91]. In addition, isoflavones are considered beneficial in the prevention of CVD. However, it has been suggested that depending on the soy intake, equol (daidzein metabolite) contributes more significantly to the reduced CV risk—equol excretors consuming more soya isoflavones presented a significantly lower cIMT and higher HDL-cholesterol concentrations than equol excretors with a lower soya intake [92]. Additionally, dried garlic powder supplementation (in the form of tablets) has been associated with the slightly lower cIMT values following three months of treatment [93].

### 4.3. Weight Loss Interventions and Bariatric Surgery

As already mentioned, obesity has undoubtedly been associated with an increased risk of premature death and an increased CVD morbidity. Furthermore, one of the approaches to reduce both obesity and the increased CV risk is weight loss, which can be achieved behaviourally, pharmacologically or surgically [94]. A 12-month weight loss of at least 5% reduced mean cIMT by 0.02 mm among patients with severe obesity. Nevertheless, although these results were not statistically significant, the authors estimated that weight loss could reduce the long-term risk of myocardial infarction and stroke rates by 3% and 4%, respectively [95]. Moreover, weight loss was easier to achieve when dietary intervention and physical activity were combined, compared with the case when only dietary intervention was implemented at the beginning (and then physical activity was introduced after 6 months)—56% vs. 44% of these two groups reduced their weight, respectively. A reduction in cIMT was positively correlated with changes in BMI, waist circumference, fat-free mas, as well as with leptin and insulin concentrations. Similar results were observed in the study by Vamvakis et al., where intensive lifestyle treatment (diet combined with physical activity) improved the cIMT values [96]. Interestingly, medical nutrition therapy provided by a registered dietitian was more beneficial than the usual care. On the other hand, in other studies, the cIMT values remained unchanged after nine months, or one year, of lifestyle intervention [97,98]. Nonetheless, as reported by Marshall et al., there was a tendency for cIMT to decrease in individuals whose Heart Health Index (HHI)—modelled following several healthy lifestyle scores—was equal to or higher than 3, compared with individuals whose HHI score was lower than 3 [98].

Bariatric surgery is an invasive therapeutic approach in the treatment of obesity. It can be performed in patients with severe and morbid obesity or in individuals with the second-degree obesity with present comorbidities. Bariatric surgery is an extremely effective treatment of weight-loss improving the metabolic status, also with regard to the CV risk. Roux-en-Y gastric bypass surgery (RYGB) significantly reduced the mean cIMT values after 12 months since the surgery in patients with type 2 diabetes or an impaired glucose tolerance, but not in individuals presenting normal glucose tolerance. It highlights the early, positive changes in the atherosclerotic plaque—which proved to be reversible—following the surgery in patients with impaired glycaemic profile or T2DM [99]. Similar results were obtained in the study by Lambert et al., where a significant reduction of the cIMT values was observed only after 1–2 months following bariatric surgery (RYGB), independent of weight loss, but associated with an early reduction in leptin concentrations [100]. Another study also confirmed the role of RYGB and sleeve gastrectomy in reducing the atherosclerotic risk by decreasing the values of cIMT after the surgery [101].

## 5. Conclusions

The cIMT measurement is essential among patients with obesity, even without the metabolic implications, and can be helpful in the diagnosis of subclinical atherosclerosis in metabolically unhealthy individuals presenting normal weight. However, it is necessary to perform further clinical investigations in order to determine a definitive relationship between the modifications in cIMT and the changes in CV. Several nutrients, e.g., folate and folic acid, vitamin K, vitamin B12, vitamin D or magnesium, have been found to positively influence the cIMT values, which could provide a potential approach to decrease the atherosclerosis risk. Moreover, it is vital to focus particularly on the role of behavioural aspects, e.g., dietary patterns, such as DASH or the Mediterranean diet, as well as on the clinical factors, including bariatric surgery, which can be effective in decreasing both the cIMT values and the atherosclerosis risk in patients with obesity.

## Data Availability

Data are available and publicly accessible. The data presented in this study are openly available in the Medline and PubMed databases and on the publisher’s website. The keywords that were used include: “intima-media thickness”, “obesity”, “bariatric surgery”, “diet”, “physical activity”, “cardiovascular risk”. All data in the text are quoted and all works used are listed in the bibliography along with doi and reference numbers.

## References

[1] Pahwa R., Jialal I. (2020). Atherosclerosis. StatPearls.

[2] Timmis A., Townsend N., Gale C.P., Torbica A., Lettino M., Petersen E.S., Mossialos E.A., Maggioni A.P., Kazakiewicz D., May H.T. (2020). European Society of Cardiology: Cardiovascular Disease Statistics 2019. Eur. Heart J..

[3] Obesity and Overweight. https://www.who.int/news-room/fact-sheets/detail/obesity-and-overweight.

[4] Xue R., Li Q., Geng Y., Wang H., Wang F., Zhang S. (2021). Abdominal obesity and risk of CVD: A dose–response meta-analysis of thirty-one prospective studies. Br. J. Nutr..

[5] Pignoli P., Tremoli E., Poli A., Oreste P., Paoletti R. (1986). Intimal plus medial thickness of the arterial wall: A direct measurement with ultrasound imaging. Circulation.

[6] Koliaki C., Liatis S., Kokkinos A. (2019). Obesity and cardiovascular disease: Revisiting an old relationship. Metabolism.

[7] Mandviwala T., Khalid U., Deswal A. (2016). Obesity and Cardiovascular Disease: A Risk Factor or a Risk Marker?. Curr. Atheroscler. Rep..

[8] Karczewski J., Śledzińska E., Baturo A., Jończyk I., Maleszko A., Samborski P., Begier-Krasińska B., Dobrowolska A. (2018). Obesity and inflammation. Eur. Cytokine Netw..

[9] Ellulu M.S., Patimah I., Khaza’Ai H., Rahmat A., Abed Y. (2017). Obesity and inflammation: The linking mechanism and the complications. Arch. Med. Sci..

[10] Amin M.N., Hussain S., Sarwar S., Moghal M.R., Das A., Hossain M.Z., Chowdhury J.A., Millat S., Islam M.S. (2019). How the association between obesity and inflammation may lead to insulin resistance and cancer. Diabetes Metab. Syndr. Clin. Res. Rev..

[11] Rychter A., Skrzypczak-Zielińska M., Zielińska A., Eder P., Souto E., Zawada A., Ratajczak A., Dobrowolska A., Krela-Kaźmierczak I. (2020). Is the Retinol-Binding Protein 4 a Possible Risk Factor for Cardiovascular Diseases in Obesity?. Int. J. Mol. Sci..

[12] Kozakova M., Natali A., Dekker J., Beck-Nielsen H., Laakso M., Nilsson P., Balkau B., Ferrannini E. (2013). RISC Investigators Insulin Sensitivity and Carotid Intima-Media Thickness: Relationship between Insulin Sensitivity and Cardiovascular Risk Study. Arter. Thromb. Vasc. Biol..

[13] Ahmad J., Ahmed F., Siddiqui M.A., Hameed B., Ahmad I. (2006). Inflammation, insulin resistance and carotid IMT in first degree relatives of north Indian type 2 diabetic subjects. Diabetes Res. Clin. Pract..

[14] Xiao Y., Xu A., Hui X., Zhou P., Li X., Zhong H., Tang W., Huang G., Zhou Z. (2013). Circulating Lipocalin-2 and Retinol-Binding Protein 4 Are Associated with Intima-Media Thickness and Subclinical Atherosclerosis in Patients with Type 2 Diabetes. PLoS ONE.

[15] Waśkiewicz A., Szcześniewska D., Szostak-Węgierek D., Kwaśniewska M., Pająk A., Stepaniak U., Kozakiewicz K., Tykarski A., Zdrojewski T., Zujko M.E. (2016). Are dietary habits of the Polish population consistent with the recommendations for prevention of cardiovascular disease?—WOBASZ II project. Kardiol. Polska.

[16] Poredos P. (2004). Intima-media thickness: Indicator of cardiovascular risk and measure of the extent of atherosclerosis. Vasc. Med..

[17] Pérez-Larraya J.G., Irimia P., Martínez-Vila E., Barba J., Guembe M.J., Varo N., Castellano J.M., Díez J. (2012). The influence of obesity on the assessment of carotid intima-media thickness. J. Clin. Ultrasound.

[18] Bots M.L., Breslau P.J., Briët E., De Bruyn A.M., Van Vliet H.H., Ouweland F.A.V.D., De Jong P.T., Hofman A., Grobbee D.E. (1992). Cardiovascular determinants of carotid artery disease. The Rotterdam Elderly Study. Hypertension.

[19] Folsom A.R., Kronmal R.A., Detrano R.C., O’Leary D.H., Bild D.E., Bluemke D.A., Budoff M.J., Liu K., Shea S., Szklo M. (2008). Coronary Artery Calcification Compared With Carotid Intima-Media Thickness in the Prediction of Cardiovascular Disease IncidenceThe Multi-Ethnic Study of Atherosclerosis (MESA). Arch. Intern. Med..

[20] Bild D.E., Bluemke D.A., Burke G.L., Detrano R., Roux A.V.D., Folsom A.R., Greenland P., Jacobs D.R., Kronmal R.A., Liu K. (2002). Multi-Ethnic Study of Atherosclerosis: Objectives and Design. Am. J. Epidemiol..

[21] Lorenz M.W., von Kegler S., Steinmetz H., Markus H.S., Sitzer M. (2006). Carotid Intima-Media Thickening Indicates a Higher Vascular Risk Across a Wide Age Range: Prospective Data from the Carotid Atherosclerosis Progression Study (CAPS). Stroke.

[22] Baigent C., Koskinas K.C., Casula M., Badimon L., Chapman M.J., Backer G.G.D., Delgado V., Ference B.A., Graham I.M., Halliday A. (2020). 2019 ESC/EAS Guidelines for the Management of Dyslipidaemias: Lipid Modification to Reduce Cardiovascular Risk. Eur. Heart J..

[23] Stein J.H., Korcarz C., Hurst R.T., Lonn E., Kendall C.B., Mohler E.R., Najjar S.S., Rembold C.M., Post W.S. (2008). Use of Carotid Ultrasound to Identify Subclinical Vascular Disease and Evaluate Cardiovascular Disease Risk: A Consensus Statement from the American Society of Echocardiography Carotid Intima-Media Thickness Task Force Endorsed by the Society for Vascular Medicine. J. Am. Soc. Echocardiogr..

[24] Touboul P., Hennerici M., Meairs S., Adams H., Amarenco P., Bornstein N., Csiba L., Ebrahim S., Hernandez R.H., Jaff M. (2012). Mannheim Carotid Intima-Media Thickness and Plaque Consensus (2004–2006–2011): An Update on Behalf of the Advisory Board of the 3rd and 4th Watching the Risk Symposium 13th and 15th European Stroke Conferences, Mannheim, Germany, 2004, and Brussels, Belgium, 2006. Cerebrovasc. Dis..

[25] Wang Z., Li W., Tian J. (2021). Gender is a determinant of carotid artery stiffness independent of age and blood pressure. Br. J. Radiol..

[26] Ojima S., Kubozono T., Kawasoe S., Kawabata T., Miyahara H., Tokushige K., Ohishi M. (2021). Gender differences in the risk factors associated with atherosclerosis by carotid intima-media thickness, plaque score, and pulse wave velocity. Heart Vessel..

[27] Simon A., Gariepy J., Chironi G., Megnien J.-L., Levenson J. (2002). Intima–media thickness: A new tool for diagnosis and treatment of cardiovascular risk. J. Hypertens..

[28] Jarauta E., Mateo-Gallego R., Bea A., Burillo E., Calmarza P., Civeira F. (2010). Carotid Intima-Media Thickness in Subjects With No Cardiovascular Risk Factors. Rev. Esp. Cardiol. (Engl. Ed.).

[29] Mil S.R., Biter L.U., Geijn G.J.M., Birnie E., Dunkelgrun M., Ijzermans J.N.M., Meulen N., Mannaerts G.H.H., Cabezas M.C. (2019). The effect of sex and menopause on carotid intima-media thickness and pulse wave velocity in morbid obesity. Eur. J. Clin. Investig..

[30] Buscemi S., Chiarello P., Buscemi C., Corleo D., Massenti M.F., Barile A.M., Rosafio G., Maniaci V., Settipani V., Cosentino L. (2017). Characterization of Metabolically Healthy Obese People and Metabolically Unhealthy Normal-Weight People in a General Population Cohort of the ABCD Study. J. Diabetes Res..

[31] Romagnolli C., Bensenor I.M., Santos I.S., Lotufo P.A., Bittencourt M.S. (2020). Impact of metabolically healthy obesity on carotid intima-media thickness—The Brazilian Longitudinal Study of Adult Health. Nutr. Metab. Cardiovasc. Dis..

[32] Farcas A.D., Vonica C.L., Golea A. (2017). Non-alcoholic fatty liver disease, bulb carotid intima-media thickness and obesity phenotypes: Results of a prospective observational study. Med. Ultrason..

[33] Jae S.Y., Franklin B., Choi Y.-H., Fernhall B. (2015). Metabolically Healthy Obesity and Carotid Intima-Media Thickness. Mayo Clin. Proc..

[34] Talavera-Garcia E., Delgado-Lista J., Garcia-Rios A., Delgado-Casado N., Gomez-Luna P., Gomez-Garduño A., Delgado F.G., Alcala-Diaz J.F., Yubero-Serrano E., Marin C. (2016). Influence of Obesity and Metabolic Disease on Carotid Atherosclerosis in Patients with Coronary Artery Disease (CordioPrev Study). PLoS ONE.

[35] Lin A., Lacy M.E., Eaton C., Correa A., Wu W.-C. (2016). Inflammatory Obesity Phenotypes, Gender Effects and Subclinical Atherosclerosis in African Americans: The Jackson Heart Study. Arterioscler. Thromb. Vasc. Biol..

[36] Polak J.F., Pencina M.J., Pencina K.M., O’Donnell C.J., Wolf P.A., D’Agostino R.B. (2011). Carotid-Wall Intima–Media Thickness and Cardiovascular Events. N. Engl. J. Med..

[37] Lorenz M.W., Markus H.S., Bots M.L., Rosvall M., Sitzer M. (2007). Prediction of Clinical Cardiovascular Events With Carotid Intima-Media Thickness: A Systematic Review and Meta-Analysis. Circulation.

[38] Willeit P., Tschiderer L., Allara E., Reuber K., Seekircher L., Gao L., Liao X., Lonn E., Gerstein H.C., Yusuf S. (2020). Carotid Intima-Media Thickness Progression as Surrogate Marker for Cardiovascular Risk: Meta-Analysis of 119 Clinical Trials Involving 100,667 Patients. Circulation.

[39] Talari H.R., Rafiee M., Farrokhian A., Raygan F., Bahmani F., Mofrad M.D., Hamidian Y., Tamtaji O.R., Karamali F., Asemi Z. (2016). The Effects of Folate Supplementation on Carotid Intima-Media Thickness and Metabolic Status in Patients with Metabolic Syndrome. Ann. Nutr. Metab..

[40] Ntaios G., Savopoulos C., Karamitsos D., Economou I., Destanis E., Chryssogonidis I., Pidonia I., Zebekakis P., Polatides C., Sion M. (2010). The effect of folic acid supplementation on carotid intima-media thickness in patients with cardiovascular risk: A randomized, placebo-controlled trial. Int. J. Cardiol..

[41] Asemi Z., Karamali M., Esmaillzadeh A. (2014). Metabolic response to folate supplementation in overweight women with polycystic ovary syndrome: A randomized double-blind placebo-controlled clinical trial. Mol. Nutr. Food Res..

[42] Qin X., Xu M., Zhang Y., Li J., Xu X., Wang X., Xu X., Huo Y. (2012). Effect of folic acid supplementation on the progression of carotid intima-media thickness: A meta-analysis of randomized controlled trials. Atherosclerosis.

[43] Appel L.J. (2004). Beyond (or back to) traditional risk factors: Preventing cardiovascular disease in patients with chronic kidney disease. Ann. Intern. Med..

[44] Devi N.H., Chaitanya V., Suchitra M.M., Rao P.V.L.N.S., Lakshmi B.V., Kumar V.S. (2018). Osteopontin, cardiovascular risk factors and carotid intima-Media thickness in chronic kidney disease. Indian J. Nephrol..

[45] Yu F.-P., Zhao Y.-C., Gu B., Hu J., Yang Y.-Y. (2015). Chronic Kidney Disease and Carotid Atherosclerosis in Patients With Acute Stroke. Neurologist.

[46] Kajitani N., Uchida H.A., Suminoe I., Kakio Y., Kitagawa M., Sato H., Wada J. (2018). Chronic kidney disease is associated with carotid atherosclerosis and symptomatic ischaemic stroke. J. Int. Med. Res..

[47] Afsar B., Yilmaz M.I., Siriopol D., Unal H.U., Saglam M., Karaman M., Gezer M., Sonmez A., Eyileten T., Aydin I. (2016). Thyroid function and cardiovascular events in chronic kidney disease patients. J. Nephrol..

[48] Su Y., Huang Y., Jiang Y., Zhu M. (2020). The Association between Serum Retinol-Binding Protein 4 Levels and Cardiovascular Events in Patients with Chronic Kidney Disease. Lab. Med..

[49] Ito S., Nagasawa T., Abe M., Mori T. (2009). Strain vessel hypothesis: A viewpoint for linkage of albuminuria and cerebro-cardiovascular risk. Hypertens. Res..

[50] Yang S.-Y., Li X.-J., Zhang W., Liu C.-Q., Zhang H.-J., Lin J.-R., Yan B., Yu Y.-X., Shi X.-L., Li C.-D. (2012). Chinese Lacto-Vegetarian Diet Exerts Favorable Effects on Metabolic Parameters, Intima-Media Thickness, and Cardiovascular Risks in Healthy Men. Nutr. Clin. Pract..

[51] Kwok T., Chook P., Qiao M., Tam L., Poon Y.K.P., Ahuja A.T., Woo J., Celermajer D.S., Woo K.S. (2012). Vitamin B-12 supplementation improves arterial function in vegetarians with subnormal vitamin B-12 status. J. Nutr. Health Aging.

[52] Petchey W.G., Hickman I.J., Duncan E., Prins J.B., Hawley C.M., Johnson D.W., Barraclough K., Isbel N.M. (2009). The role of 25-hydroxyvitamin D deficiency in promoting insulin resistance and inflammation in patients with Chronic Kidney Disease: A randomised controlled trial. BMC Nephrol..

[53] Asemi Z., Karamali M., Esmaillzadeh A. (2014). Effects of calcium–vitamin D co-supplementation on glycaemic control, inflammation and oxidative stress in gestational diabetes: A randomised placebo-controlled trial. Diabetologia.

[54] Asemi Z., Raygan F., Bahmani F., Rezavandi Z., Talari H.R., Rafiee M., Poladchang S., Mofrad M.D., Taheri S., Mohammadi A.A. (2016). The effects of vitamin D, K and calcium co-supplementation on carotid intima-media thickness and metabolic status in overweight type 2 diabetic patients with CHD. Br. J. Nutr..

[55] Manson J.E., Cook N.R., Lee I.-M., Christen W., Bassuk S.S., Mora S., Gibson H., Gordon D., Copeland T., D’Agostino D. (2019). Vitamin D Supplements and Prevention of Cancer and Cardiovascular Disease. N. Engl. J. Med..

[56] Kurnatowska I., Grzelak P., Masajtis-Zagajewska A., Kaczmarska M., Stefańczyk L., Vermeer C., Maresz K., Nowicki M. (2015). Effect of vitamin K2 on progression of atherosclerosis and vascular calcification in nondialyzed patients with chronic kidney disease stages 3–5. Pol. Arch. Intern. Med..

[57] Li S., Na L., Li Y., Gong L., Yuan F., Niu Y., Zhao Y., Sun C. (2013). Long-term calcium supplementation may have adverse effects on serum cholesterol and carotid intima-media thickness in postmenopausal women: A double-blind, randomized, placebo-controlled trial. Am. J. Clin. Nutr..

[58] Reid I.R., Mason B., Horne A., Ames R., Clearwater J., Bava U., Orr-Walker B., Wu F., Evans M.C., Gamble G.D. (2002). Effects of Calcium Supplementation on Serum Lipid Concentrations in Normal Older Women: A Randomized Controlled Trial. Am. J. Med..

[59] Bostick R.M., Fosdick L., Grandits G.A., Grambsch P., Gross M., Louis T.A. (2000). Effect of Calcium Supplementation on Serum Cholesterol and Blood Pressure: A Randomized, Double-blind, Placebo-Controlled, Clinical Trial. Arch. Fam. Med..

[60] Gannagé-Yared M.H., Azoury M., Mansour I., Baddoura R., Halaby G., Naaman R. (2003). Effects of a short-term calcium and vitamin D treatment on serum cytokines, bone markers, insulin and lipid concentrations in healthy post-menopausal women. J. Endocrinol. Investig..

[61] Reid I.R. (2013). Cardiovascular Effects of Calcium Supplements. Nutrients.

[62] Talari H.R., Zakizade M., Soleimani A., Bahmani F., Ghaderi A., Mirhosseini N., Eslahi M., Babadi M., Mansournia M.A., Asemi Z. (2019). Effects of magnesium supplementation on carotid intima–media thickness and metabolic profiles in diabetic haemodialysis patients: A randomised, double-blind, placebo-controlled trial. Br. J. Nutr..

[63] Silva A.P. (2014). Magnesium and Mortality in Patients with Diabetes and Early Chronic Kidney Disease. J. Diabetes Metab..

[64] Tzanakis I., Virvidakis K., Tsomi A., Mantakas E., Girousis N., Karefyllakis N., Papadaki A., Kallivretakis N., Mountokalakis T. (2004). Intra- and extracellular magnesium levels and atheromatosis in haemodialysis patients. Magnes. Res..

[65] Chacko S.A., Sul J., Song Y., Li X., Leblanc J., You Y., Butch A., Liu S. (2010). Magnesium supplementation, metabolic and inflammatory markers, and global genomic and proteomic profiling: A randomized, double-blind, controlled, crossover trial in overweight individuals. Am. J. Clin. Nutr..

[66] Gardener H., Wright C.B., Gu Y., Demmer R.T., Boden-Albala B., Elkind M.S.V., Sacco R.L., Scarmeas N. (2011). Mediterranean-style diet and risk of ischemic stroke, myocardial infarction, and vascular death: The Northern Manhattan Study. Am. J. Clin. Nutr..

[67] Rychter A.M., Ratajczak A.E., Zawada A., Dobrowolska A., Krela-Kaźmierczak I. (2020). Non-Systematic Review of Diet and Nutritional Risk Factors of Cardiovascular Disease in Obesity. Nutrients.

[68] Gardener H., Wright C., Cabral D., Scarmeas N., Gu Y., Cheung K., Elkind M.S., Sacco R.L., Rundek T. (2014). Mediterranean diet and carotid atherosclerosis in the Northern Manhattan Study. Atherosclerosis.

[69] Murie-Fernandez M., Irimia P., Toledo E., Martínez-Vila E., Buil-Cosiales P., Serrano-Martínez M., Ruíz-Gutierrez V., Ros E., Estruch R., Martínez-González M. (2011). Ángel Carotid intima-media thickness changes with Mediterranean diet: A randomized trial (PREDIMED-Navarra). Atherosclerosis.

[70] Sala-Vila A., Romero-Mamani E.-S., Gilabert R., Núñez I., De La Torre R., Corella L., Ruiz-Gutiérrez V., López-Sabater M.C., Pintó X., Rekondo J. (2014). Changes in Ultrasound-Assessed Carotid Intima-Media Thickness and Plaque With a Mediterranean Diet: A Substudy of the PREDIMED Trial. Arter. Thromb. Vasc. Biol..

[71] Guasch-Ferré M., Salas-Salvadó J., Ros E., Estruch R., Corella D., Fitó M., Martínez-González M., Arós F., Gómez-Gracia E., Fiol M. (2017). The PREDIMED trial, Mediterranean diet and health outcomes: How strong is the evidence?. Nutr. Metab. Cardiovasc. Dis..

[72] Estruch R., Ros E., Salas-Salvadó J., Covas M.-I., Corella D., Arós F., Gómez-Gracia E., Ruiz-Gutiérrez V., Fiol M., Lapetra J. (2013). Primary Prevention of Cardiovascular Disease with a Mediterranean Diet. N. Engl. J. Med..

[73] Ros E., Martínez-González M.A., Estruch R., Salas-Salvadó J., Fitó M., Martínez J.A., Corella D. (2014). Mediterranean Diet and Cardiovascular Health: Teachings of the PREDIMED Study. Adv. Nutr..

[74] Ros E. (2017). The PREDIMED study. Endocrinol. Diabetes Nutr..

[75] Maiorino M.I., Bellastella G., Petrizzo M., Scappaticcio L., Giugliano D., Esposito K. (2016). Mediterranean diet cools down the inflammatory milieu in type 2 diabetes: The MÉDITA randomized controlled trial. Endocrine.

[76] Maiorino M.I., Bellastella G., Petrizzo M., Gicchino M., Caputo M., Giugliano D., Esposito K. (2017). Effect of a Mediterranean diet on endothelial progenitor cells and carotid intima-media thickness in type 2 diabetes: Follow-up of a randomized trial. Eur. J. Prev. Cardiol..

[77] da Luz P., Coimbra S., Favarato D., Albuquerque C., Mochiduky R., Rochitte C., Hojaij E., Gonsalves C., Laurindo F. (2014). Coronary artery plaque burden and calcium scores in healthy men adhering to long-term wine drinking or alcohol abstinence. Braz. J. Med. Biol. Res..

[78] Siervo M., Lara J., Chowdhury S., Ashor A., Oggioni C., Mathers J.C. (2015). Effects of the Dietary Approach to Stop Hypertension (DASH) diet on cardiovascular risk factors: A systematic review and meta-analysis. Br. J. Nutr..

[79] Dos Santos K., Moreira T.M., Belfort G.P., Silva C.F.D.M.D., Padilha P.D.C., De Barros D.C., Saunders C. (2019). Adaptação da dieta DASH (Dietary Approaches to Stop Hypertension) para cuidado nutricional no período pós-parto, no âmbito da Atenção Básica. Rev. Bras. Epidemiol..

[80] Maddock J., Ziauddeen N., Ambrosini G.L., Wong A., Hardy R., Ray S. (2018). Adherence to a Dietary Approaches to Stop Hypertension (DASH)-type diet over the life course and associated vascular function: A study based on the MRC 1946 British birth cohort. Br. J. Nutr..

[81] De Oliveira P.A., Kovacs C., Moreira P., Magnoni D., Saleh M.H., Faintuch J. (2017). Unsaturated Fatty Acids Improve Atherosclerosis Markers in Obese and Overweight Non-diabetic Elderly Patients. Obes. Surg..

[82] Angerer P., Kothny W., Störk S., von Schacky C. (2002). Effect of dietary supplementation with ω-3 fatty acids on progression of atherosclerosis in carotid arteries. Cardiovasc. Res..

[83] Virtanen J.K., Mursu J., Tuomainen T.-P., Voutilainen S. (2014). Dietary Fatty Acids and Risk of Coronary Heart Disease in Men: The Kuopio Ischemic Heart Disease Risk Factor Study. Arter. Thromb. Vasc. Biol..

[84] Chen C.-Y., Huang W.-S., Chen H.-C., Chang C.-H., Lee L.-T., Chen H.-S., Kang Y.-D., Chie W.-C., Jan C.-F., Wang W.-D. (2020). Effect of a 90 g/day low-carbohydrate diet on glycaemic control, small, dense low-density lipoprotein and carotid intima-media thickness in type 2 diabetic patients: An 18-month randomised controlled trial. PLoS ONE.

[85] Petersen K., Keogh J., Lister N., Clifton P. (2018). Dietary quality and carotid intima media thickness in type 1 and type 2 diabetes: Follow-up of a randomised controlled trial. Nutr. Metab. Cardiovasc. Dis..

[86] Petersen K.S., Clifton P.M., Blanch N., Keogh J.B. (2015). Effect of improving dietary quality on carotid intima media thickness in subjects with type 1 and type 2 diabetes: A 12-mo randomized controlled trial. Am. J. Clin. Nutr..

[87] Chiavaroli L., Mirrahimi A., Ireland C., Mitchell S., Sahye-Pudaruth S., Coveney J., Olowoyeye O., Patel D., De Souza R.J., Augustin L.S. (2017). Cross-sectional associations between dietary intake and carotid intima media thickness in type 2 diabetes: Baseline data from a randomised trial. BMJ Open.

[88] Zhu Y., Zhang Y., Ling W., Feng D., Wei X., Yang C., Ma J. (2011). Fruit Consumption Is Associated with Lower Carotid Intima-Media Thickness and C-Reactive Protein Levels in Patients with Type 2 Diabetes Mellitus. J. Am. Diet. Assoc..

[89] Blekkenhorst L.C., Bondonno C.P., Lewis J.R., Woodman R., Devine A., Bondonno N.P., Lim W.H., Zhu K., Beilin L.J., Thompson P.L. (2018). Cruciferous and Total Vegetable Intakes Are Inversely Associated With Subclinical Atherosclerosis in Older Adult Women. J. Am. Heart Assoc..

[90] Bondonno C.P., Blekkenhorst L.C., Prince R.L., Ivey K.L., Lewis J.R., Devine A., Woodman R.J., Lundberg J.O., Croft K.D., Thompson P.L. (2017). Association of Vegetable Nitrate Intake With Carotid Atherosclerosis and Ischemic Cerebrovascular Disease in Older Women. Stroke.

[91] Zou Z.-Y., Xu X.-R., Lin X.-M., Zhang H.-B., Xiao X., Ouyang L., Huang Y.-M., Wang X., Liu Y.-Q. (2014). Effects of lutein and lycopene on carotid intima–media thickness in Chinese subjects with subclinical atherosclerosis: A randomised, double-blind, placebo-controlled trial. Br. J. Nutr..

[92] Cai Y., Guo K., Chen C., Wang P., Zhang B., Zhou Q., Mei F., Su Y. (2012). Soya isoflavone consumption in relation to carotid intima–media thickness in Chinese equol excretors aged 40–65 years. Br. J. Nutr..

[93] Mahdavi-Roshan M., Zahedmehr A., Mohammad-Zadeh A., Sanati H.-R., Shakerian F., Firouzi A., Kiani R., Nasrollahzadeh J. (2013). Effect of garlic powder tablet on carotid intima-media thickness in patients with coronary artery disease: A Preliminary Randomized Controlled Trial. Nutr. Health.

[94] Blüher M. (2019). Obesity: Global epidemiology and pathogenesis. Nat. Rev. Endocrinol..

[95] Cooper J.N., Columbus M.L., Shields K.J., Asubonteng J., Meyer M.L., Sutton-Tyrrell K., Goodpaster B.H., Delany J.P., Jakicic J.M., Barinas-Mitchell E. (2012). Effects of an intensive behavioral weight loss intervention consisting of caloric restriction with or without physical activity on common carotid artery remodeling in severely obese adults. Metabolism.

[96] Vamvakis A., Gkaliagkousi E., Lazaridis A., Grammatikopoulou M.G., Triantafyllou A., Nikolaidou B., Koletsos N., Anyfanti P., Tzimos C., Zebekakis P. (2020). Impact of Intensive Lifestyle Treatment (Diet Plus Exercise) on Endothelial and Vascular Function, Arterial Stiffness and Blood Pressure in Stage 1 Hypertension: Results of the HINTreat Randomized Controlled Trial. Nutrients.

[97] Elkoustaf R.A., Aldaas O.M., Batiste C.D., Mercer A., Robinson M., Newton D., Burchett R., Cornelius C., Patterson H., Ismail M.H. (2019). Lifestyle Interventions and Carotid Plaque Burden: A Comparative Analysis of Two Lifestyle Intervention Programs in Patients with Coronary Artery Disease. Perm. J..

[98] Marshall D., Walizer E., Vernalis M. (2011). The Effect of a One-Year Lifestyle Intervention Program on Carotid Intima Media Thickness. Mil. Med..

[99] Lundby-Christensen L., Tarnow L., Hansen D.L., Worm D., Naver L.S., Hvolris L.E., Wiinberg N., Vaag A.A., Almdal T.P. (2014). Carotid intima-media thickness is reduced 12 months after gastric bypass surgery in obese patients with type 2 diabetes or impaired glucose tolerance. J. Diabetes Complicat..

[100] Lambert G., Lima M.M.D.O., Felici A.C., Pareja J.C., Vasques A.C.J., Novaes F.S., Rodovalho S., Hirsch F.F.P., Matos-Souza J.R., Chaim É.A. (2017). Early Regression of Carotid Intima-Media Thickness after Bariatric Surgery and Its Relation to Serum Leptin Reduction. Obes. Surg..

[101] Gómez-Martin J.M., Aracil E., Galindo J., Escobar-Morreale H.F., Balsa J.A., Botella-Carretero J.I. (2017). Improvement in cardiovascular risk in women after bariatric surgery as measured by carotid intima-media thickness: Comparison of sleeve gastrectomy versus gastric bypass. Surg. Obes. Relat. Dis..

